# Climate change winners and losers: The effects of climate change on five palm species in the Southeastern United States

**DOI:** 10.1002/ece3.6697

**Published:** 2020-09-01

**Authors:** Christopher J. Butler, Matt Larson

**Affiliations:** ^1^ Department of Biology University of Central Oklahoma Edmond OK USA

**Keywords:** climate change, distribution, ecological niche model, MaxEnt, palm

## Abstract

Palms (Arecaceae) are a relatively speciose family and provide materials for food, construction, and handicraft, especially in the tropics. They are frequently used as paleo‐indicators for megathermal climates, and therefore, it is logical to predict that palms will benefit from predicted warmer temperatures under anthropogenic climate change. We created species distribution models to explore the projected ranges of five widespread southeastern North American palm species (*Rhapidophyllum hystrix*, *Sabal etonia*, *Sabal minor*, *Sabal palmetto*, and *Serenoa repens*) under four climate change scenarios through 2070. We project that the amount of habitat with >50% suitability for *S. etonia* will decline by a median of 50% by 2070, while the amount of habitat with >50% suitability *S. minor* will decline by a median of 97%. In contrast, the amount of suitable habitat for *Rhapidophyllum hystrix* will remain stable, while the amount of suitable habitat for *Serenoa repens* will slightly increase. The projected distribution for *S. palmetto* will increase substantially, by a median of approximately 21% across all scenarios. The centroid of the range of each species will shift generally north at a median rate of 23.5 km/decade. These five palm species have limited dispersal ability and require a relatively long time to mature and set fruit. Consequently, it is likely that the change in the distribution of these palms will lag behind the projected changes in climate. However, Arecaceae can modify physiological responses to heat and drought, which may permit these palms to persist as local conditions become increasingly inappropriate. Nonetheless, this plasticity is unlikely to indefinitely prevent local extinctions.

## Graphical abstract

Palms (Arecaceae) are widely used as paleo‐indicators of megathermal climates, and it would be expected that the their range should increase with anthropogenic climate change. We examined the potential effects of climate change by 2070 on five widespread palm species in the southeastern United States and find that the amount of suitable habitat for two species will decline substantially, one species will remain essentially unchanged, and two species will benefit.

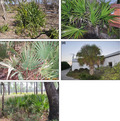

## 1 INTRODUCTION

Anthropogenic climate change has caused widespread changes in biological communities, with ongoing shifts in distribution, phenology, ecophysiology, and community interactions (Blois, Zarnetske, Fitzpatrick, & Finnegan, 2013; Butler, 2019; Parmesan & Hanley, 2015; Poloczanska et al., 2013). These changes have been linked with the spread of diseases (Semenza & Menne, 2009; Wu, Lu, Zhou, Chen, & Xu, 2016), invasive species (Stephens, Dantzler‐Kyer, Patten, & Souza, 2019; Walther et al., 2009), and a reduction in the amount of suitable habitat for species (Sekercioglu, Schneider, Fay, & Loarie, 2008; Thuiller et al., 2006). In the fynbos of South Africa, for example, Slingsby et al. (2017) found that changes in climate, fire, and invasive species were causing a decline in plant biodiversity, with the decline attributed largely to an increase in the number of hot, dry days postfire.

One plant family that could be affected by climate change is the palms (Arecaceae). Palms are largely tropical and subtropical, with the greatest diversity near the equator (Dransfield et al., 2008; Henderson, Galeano, & Bernal, 1995). The taxonomy of palms is well studied (Govaerts, Dransfield, & Zona, 2020; Harley, 2006), and many facets of their ecology, evolution, and biogeography are well studied (Baker & Couvreur, 2013; Eiserhardt, Svenning, Kissling, & Balslev, 2011; Henderson, 2002; Kissling et al., 2012). Palms are a relatively speciose family with more than 2,500 species and provide materials for construction, food, and handicraft, particularly in tropical regions (e.g., Valois‐Cuesta, Martínez Ruiz, Rentería Cuesta, & Panesso Hinestroza, 2013). For example, the IUCN estimates that oil palm plantations (*Elaeis guineensis*) cover more than 18.7 million hectares across 43 countries (Meijaard, Garcia‐Ulloa, & Sheil, 2018). Palms can also be important keystone species in the tropics (e.g., Blach‐Overgaard, Svenning, & Balslev, 2009; Voeks, 2002).

Palms are frequently employed as paleo‐indicators for megathermal (i.e., tropical) climates (e.g., Greenwood & Wing, 1995; Pross et al., 2012). A combination of climate and dispersal ability appears to be the primary factors that determine palm species richness at both the continental and global scales (Blach‐Overgaard, Kissling, Dransfield, Balslev, & Svenning, 2013; Eiserhardt et al., 2011). For instance, extinction rates in palms with megafaunal fruit in the western hemisphere have increased since the beginning of the Quaternary period, approximately 2.6 mya, due to a combination of climate oscillations and habitat fragmentation, as well as the loss of megafauna (Onstein et al., 2018).

There is some evidence that some palm species are expanding their range during recent decades. The dwarf palmetto, *S. minor*, has extended its range in Oklahoma (Butler, Curtis, McBride, Arbour, & Heck, 2011) and North Carolina (Tripp & Dexter, 2006), and individuals at the northwestern extreme of its range are undergoing a rapid population increase (Butler & Tran, 2017). The California fan palm *Washingtonia filifera* and the non‐native *Phoenix dactylifera* have begun colonizing Death Valley Springs in California (Holmquest, Schmidt‐Gengenbach, & Slaton, 2011). Chinese windmill palms (*Trachycarpus fortunei*) are gradually invading forests in Italy and Switzerland (Fehr & Burga, 2016; Walther et al., 2007).

Although there are a few studies documenting range shifts in palms, relatively little research has focused on the projected effect of climate change on palms. It has been suggested that African palms could be particularly vulnerable to anthropogenic climate change (Blach‐Overgaard et al., 2009), with up to 87% of all species negatively impacted (Blach‐Overgaard, Balslev, & Dransfield, 2015), although the near‐term potential for extinction is considerably lower than for many other plant species (Cosiaux et al., 2018). In contrast, climatic changes are forecast to increase the extent of potentially suitable areas for commercially grown date palms (*Phoenix dactylifera*) in Iran, where as much as 61 million ha are projected to become suitable for date production by 2050 (Shabani, Kumar, & Taylor, 2014).

Within the continental United States, there are fourteen native palm species: *Acoelorraphe wrightii*, *Coccothrinax argentata*, *Pseudophoenix sargentii* subsp. *sargentii*, *Rhapidophyllum hystrix*, *Roystonia regia*, *Sabal etonia*, *S. mexicana*, *S. miamiensis*, *S. minor*, *S. palmetto*, *Serenoa repens*, *Thrinax morrisii*, *T. radiata*, and *Washingtonia filifera* (Henderson et al., 1995). Five of these species are widespread in the southeastern United States, including *Rhapidophyllum hystrix, S. etonia, S. minor, S. palmetto,* and *Serenoa repens*. Large numbers of palms are commercially grown for ornamental horticulture in Florida and Texas (Broschat, Meerow, & Elliott, 2017) and four of these palm species are widely planted outside their native range, although *S. etonia* is seldom observed at commercial nurseries (pers. obs.). One species, *Serenoa repens*, is particularly commercially valuable, as it is one of the top three herbaceous dietary supplements in the United States (Jaiswal et al., 2019), generating sales of approximately $23 million USD during 2015 (Gafner & Baggett, 2017). Additionally, *Serenoa repens* is considered a keystone species (Carrington & Mullahey, 2006), with more than 200 vertebrate using it for foraging, cover, or nesting (Maehr & Layne, 1996).

Despite the importance of palms to the ecology and economy of the southeastern United States, the effects of anthropogenic climate change on the distribution of these species have not yet been investigated. Our goal was to identify the bioclimatic variables that determine the niches of these five widespread palm species in the southeastern United States. We then projected the spatial extent of these variables under multiple climate change scenarios for 2050 and 2070, in order explore how the distribution of these species might be affected by anthropogenic climate change.

## 2 MATERIALS AND METHODS

**Figure 1:**
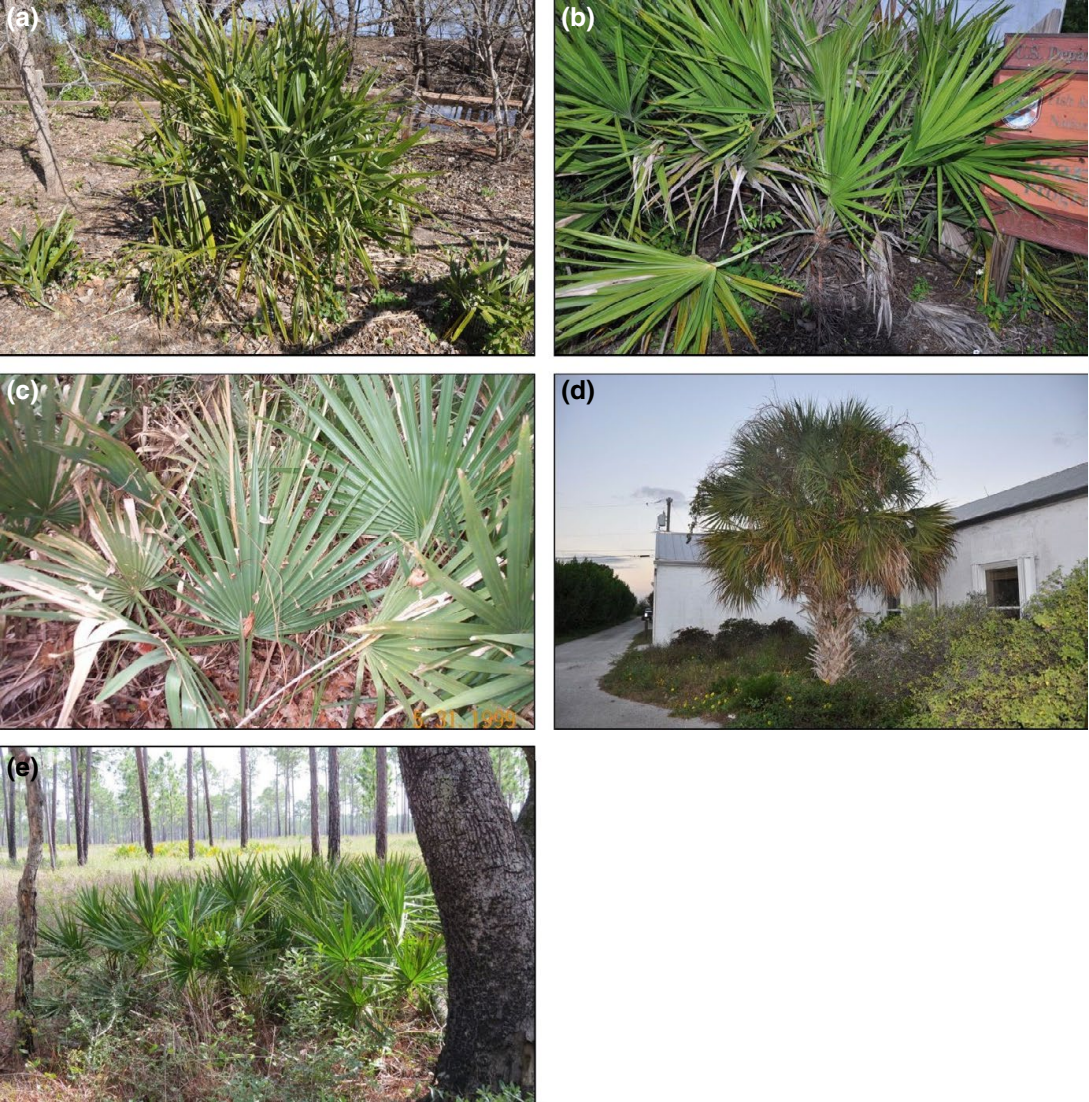
The five palm species whose distribution we examined included *Rhapidophyllum hystrix* (a), *Sabal etonia* (b), *Sabal minor* (c), *Sabal palmetto* (d), and *Serenoa repens* (e)

We modeled the current and projected ranges of five palm species: *Rhapidophyllum hystrix, Sabal etonia S. minor, S. palmetto,* and *Serenoa repens* (Phillips, Anderson, & Schapire, 2006; Phillips, Dudik, & Schapire, 2004; Figure 1). We downloaded records of these five species from the Global Biodiversity Information Facility (https://www.gbif.org/) and combined them with undigitized herbarium records from Cornell and the New York Botanical Gardens. We followed the procedures outlined in Butler, Stanila, and Iverson (2016) for data processing and model building. We eliminated duplicates and records from outside the native range and resampled the locality data to one record per 25 km^2^. We downloaded elevation and 19 bioclimatic variables from WorldClim (Hijmans, Cameron, Parra, Jones, & Jarvis, 2005; http://www.worldclim.org/) at a resolution of 2.5 arc‐minutes (25 km^2^; Table 1). We downloaded rasters of organic matter, pH, and available water content from the STATSGO2 dataset (http://websoilsurvey.nrcs.usda.gov). The spatial extent of the analysis can influence several aspects of the modeling process (Barve et al., 2011; Merow, Smith, & Silander, 2013), and it is recommended that the ecology and the dispersal abilities of the organisms be considered when building models. Since many of these palms are planted far outside their native range (e.g., *Sabal palmetto* will grow unprotected in Oklahoma City, Oklahoma, and *Sabal minor* will apparently survive in Manhattan, NY; *pers. obs*.), the spatial extent of the variables was set to the area from extreme southern Texas north to the southern third of Canada and east to the Atlantic Ocean. We followed the procedure outlined by Butler et al. (2016) and only included the variables with the most useful predictive information (i.e., the highest gain when used in isolation), as well as the variables that provided unique predictive information. As regularization multipliers (*β*) are an important component of model prediction and complexity (Moreno‐Amat et al., 2015), we used the regularization approach implemented in ENMtools (Warren, Matzke, & Cardillo, 2019) and small sample corrected variant of Akaike's information criterion (AICc) scores were used to evaluate models (Warren & Seifert, 2011) using all possible combinations of the variables that did not exhibit high multicollinearity (e.g., |*r*| < .8). We used 10,000 background points, with 70% of occurrence records used for training, and 30% used for model validation. We plotted sensitivity versus 1 ‐ specificity to created receiver operating characteristic (ROC), and 10‐fold cross‐validation AUC (area under the curve) scores were used to evaluate the accuracy of the resulting model. We used AICc scores and model weights in conjunction with AUC scores to determine the models that best describe the current distributions of the five palm species.

**Table 1:** The bioclimatic and soil variables examined in this study

Variable	Definition
AWC	Available water content
BIO 1	Annual mean temperature
BIO 2	Mean diurnal range (Mean of monthly [max temp ‐ min temp])
BIO 3	Isothermality (BIO 2/ BIO 7) * 100
BIO 4	Temperature seasonality (standard deviation * 100)
BIO 5	Max temperature of warmest month
BIO 6	Min temperature of coldest month
BIO 7	Temperature annual range (BIO 5 ‐ BIO 6)
BIO 8	Mean temperature of wettest quarter
BIO 9	Mean temperature of driest quarter
BIO 10	Mean temperature of warmest quarter
BIO 11	Mean temperature of coldest quarter
BIO 12	Annual precipitation
BIO 13	Precipitation of wettest month
BIO 14	Precipitation of driest month
BIO 15	Precipitation seasonality (coefficient of variation)
BIO 16	Precipitation of wettest quarter
BIO 17	Precipitation of driest quarter
BIO 18	Precipitation of warmest quarter
BIO 19	Precipitation of coldest quarter
OM	Organic matter
pH	pH
Elevation	Elevation above sea level

We projected the potential future distribution of *Rhapidophyllum hystrix*, *Sabal etonia, S. minor, S. palmetto*, and *Serenoa repens* at 2.5 arc‐minutes (25 km^2^) using the model that best predicted the current distribution of each species in conjunction with future climate conditions for 2050 and 2070 using the IPCC 5 data from WorldClim (Hijmans et al., 2005). Four IPCC scenarios were evaluated, including RCP 2.6, RCP 4.5, RCP 6.0, and RCP 8.5, which differed in the amount of carbon dioxide added to the atmosphere over the 21st century (Moss 2010) using 11 different general circulation models downloaded from WorldClim (BCC‐CSM1‐1, CCSM4, GISS‐E2‐R, HadGEM2‐AO, HadGEM2‐ES, IPSL‐CM5A‐LR, MIROC‐ESM‐CHEM, MIROC‐ESM, MIROC5, MRI‐CGCM3, and NorESM1‐M). We employed model averaging to create models of projected suitability under each RCP scenario for 2050 and the 2070. We classified the model results into five bands of suitability, following Butler et al. (2016): 0%–10% suitable, 10%–20% suitable, 20%–35% suitable, 35%–50% suitable, and >50% suitable. Response curves were generated for the variables in the top models to identify the range of values where suitability was >50%.

## 3 RESULTS

**Figure 2:**
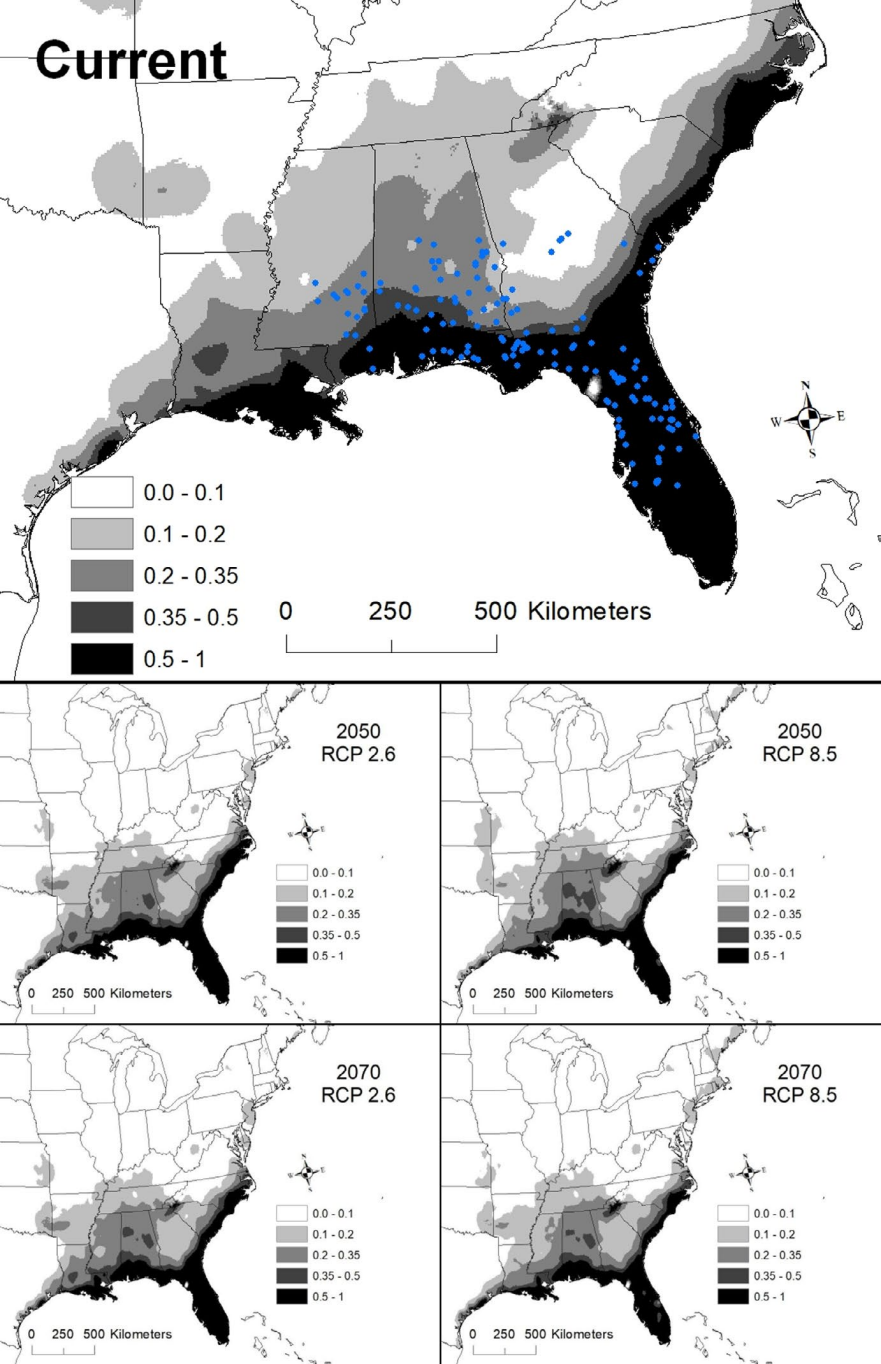
The modeled current and future distributions for *Rhapidophyllum hystrix*. The legend shows the probability of occurrence, with the darkest shade representing >0.5 probability. Blue circles represent sites where *R. hystrix* (*n = *149) were located

The best model for *Rhapidophyllum hystrix* (i.e., with the lowest AICc score) included the variables elevation, mean temperature of coldest quarter (BIO 11), precipitation of wettest month (BIO 13), and precipitation of warmest quarter (BIO 18; Table 2). The AUC for this model was 0.978 ± 0.003. Areas with suitability >50% had an elevation between 11 and 82 m a.s.l., annual mean temperature of 10.6–11.5°C, precipitation of the wettest month of 16.6–22.1 cm, and precipitation of the warmest quarter exceeding 44.8 cm. Currently suitable areas range from Mississippi east to Florida and north along the Atlantic coast to North Carolina, with discontinuous areas of potentially suitable conditions occurring in Louisiana and coastal Texas (Figure 2).

**Table 2:** The top model runs for each species, showing the variables that best explain the distribution

Species	Variables	Log likelihood	AIC_c_ score	ΔAIC_c_	wAIC_c_	Mean AUC	*β*
*Rhapidophyllum hystrix*	BIO 11, BIO 13, BIO 18, Elevation	−1,495.41	3,040.09	0	1.00	0.98	3.0
*Sabal etonia*	BIO 8, BIO 15, BIO 16, BIO 18	−729.72	1,472.55	0	0.42	0.99	1.0
	BIO 11, BIO 15, BIO 18	−725.08	1,473.22	0.67	0.30	0.99	4.0
	BIO 8, BIO 15, BIO 16	−732.08	1,474.93	2.38	0.13	0.99	0.5
	BIO 6, BIO 15, BIO 18	−727.92	1,476.30	3.76	0.07	0.99	3.0
*Sabal minor*	BIO 7, BIO 10, BIO 11, Elevation	−5,233.29	10,611.64	0	0.98	0.95	4.0
*Sabal palmetto*	BIO 8, BIO 11, BIO 13, BIO 18	−1,315.08	2,642.78	0	0.72	0.99	3.0
	BIO 11, BIO 16, BIO 18	−1,317.58	2,645.61	2.83	0.17	0.98	4.0
*Serenoa repens*	BIO 6, BIO 8, BIO 18, pH	−2,845.05	5,704.49	0	0.58	0.98	4.0
	BIO 11, BIO 18, pH	−2,847.18	5,706.66	2.16	0.20	0.98	1.0
	BIO 8, BIO 11, BIO 16, BIO 18, pH	−2,844.63	5,707.89	3.39	0.11	0.98	4.0
	BIO 8, BIO 11, BIO 18, pH	−2,845.76	5,708.02	3.53	0.10	0.98	2.0

Note

The natural log of probability of the data present in the model is given by the log likelihood. AICc is a small‐sampled corrected AIC score; only models that are within four units of the top AICc model are shown. Delta AICc is the difference between the AICc score for a model and the lowest AICc score. The model weight (wAICc) is the relative likelihood for each model, divided by the total relative likelihood for all models that were considered. AUC (area under the curve) is a metric for evaluating the accuracy of the model. The regularization multiplier is given by *β*.

**Figure 3:**
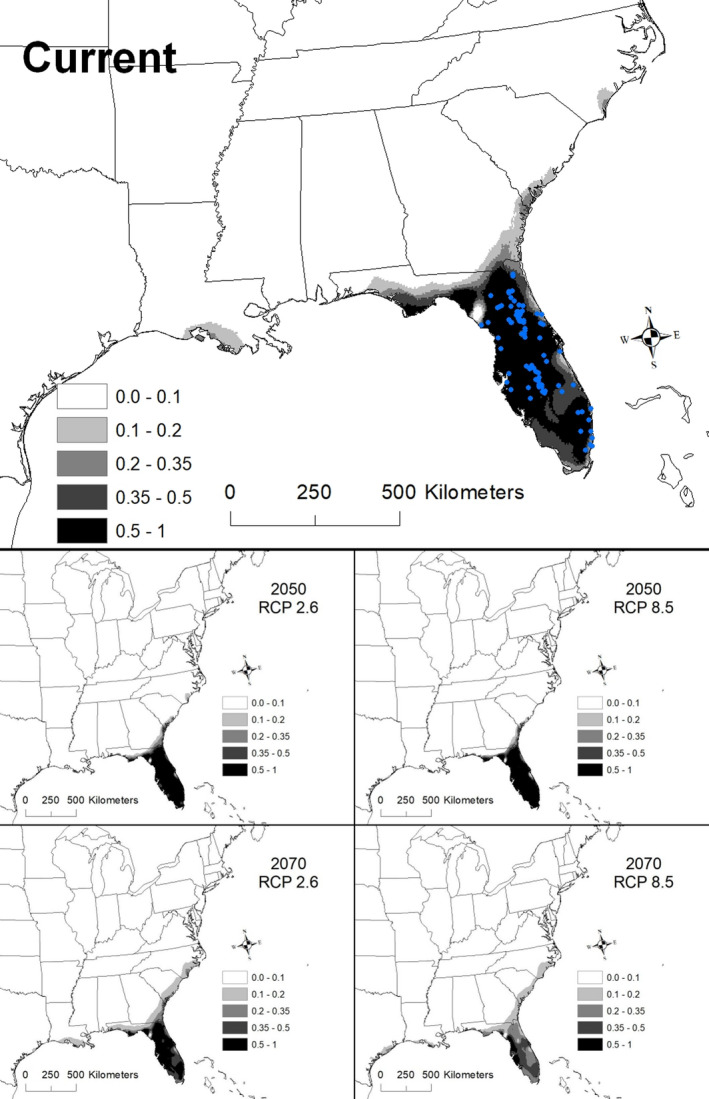
The modeled current and future distributions for *Sabal etonia*. The legend shows the probability of occurrence, with the darkest shade representing >0.5 probability. Blue circles represent sites where *S. etonia* (*n = *86) were located

The best model for *Sabal etonia* included mean temperature of wettest quarter (BIO 8), precipitation seasonality (BIO 15), precipitation of the wettest quarter (BIO 16), and precipitation of warmest quarter (BIO 18; Table 2). The AUC for this model was 0.992 ± 0.001. There was also some model support for minimum temperature of coldest month (BIO 6) and mean temperature of coldest quarter (BIO 11). Areas with suitability >50% had a mean temperature of the wettest quarter of 26.8–27.7°C, moderate precipitation seasonality (the coefficient of variation ranged from 39 to 50), precipitation of the wettest quarter of 50.6–59.0 cm, and precipitation of the warmest quarter of 50.3–57.8 cm. Areas that are currently shown as >50% suitability are restricted to Florida (Figure 3).

**Figure 4:**
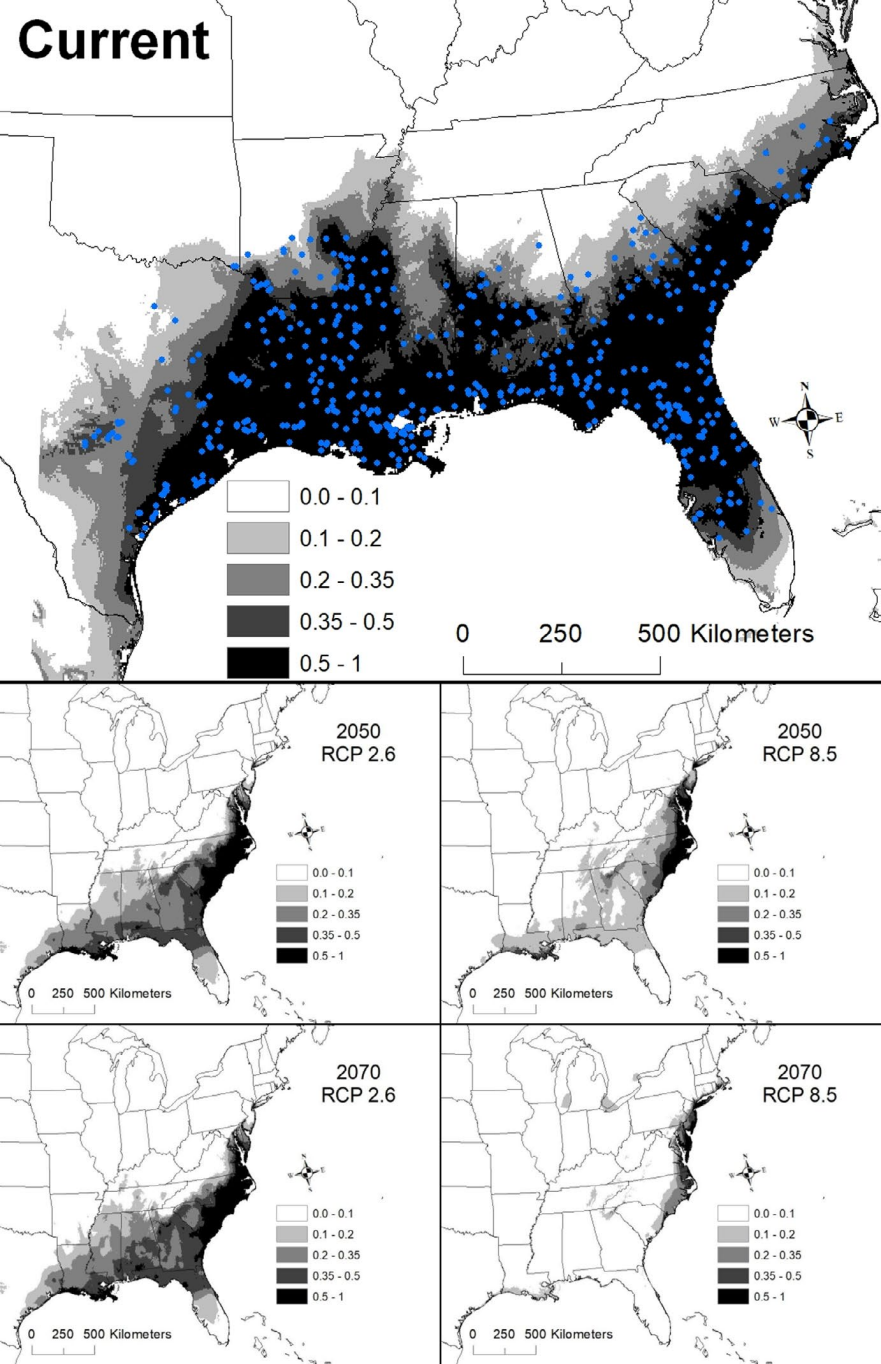
The modeled current and future distributions for *Sabal minor*. The legend shows the probability of occurrence, with the darkest shade representing >0.5 probability. Blue circles represent sites where *S. minor* (*n = *487) were located

The best model for *Sabal minor* included temperature annual range (BIO 7), mean temperature of warmest quarter (BIO 10), mean temperature of coldest quarter (BIO 11), and elevation (Table 2). The AUC for this model was 0.946 ± 0.007. Areas with suitability >50% had a temperature annual range (maximum temperature of warmest month – minimum temperature of coldest month) of 24.2–31.2°C, a mean temperature of warmest quarter from 26.5 to 27.4°C, mean temperature of coldest quarter from 9.6 to 17.2°C, and an elevation less than 67 m a. s. l. Areas with >50% suitability extended from southern Texas to southern Arkansas east to North Carolina and Florida (Figure 4).

**Figure 5:**
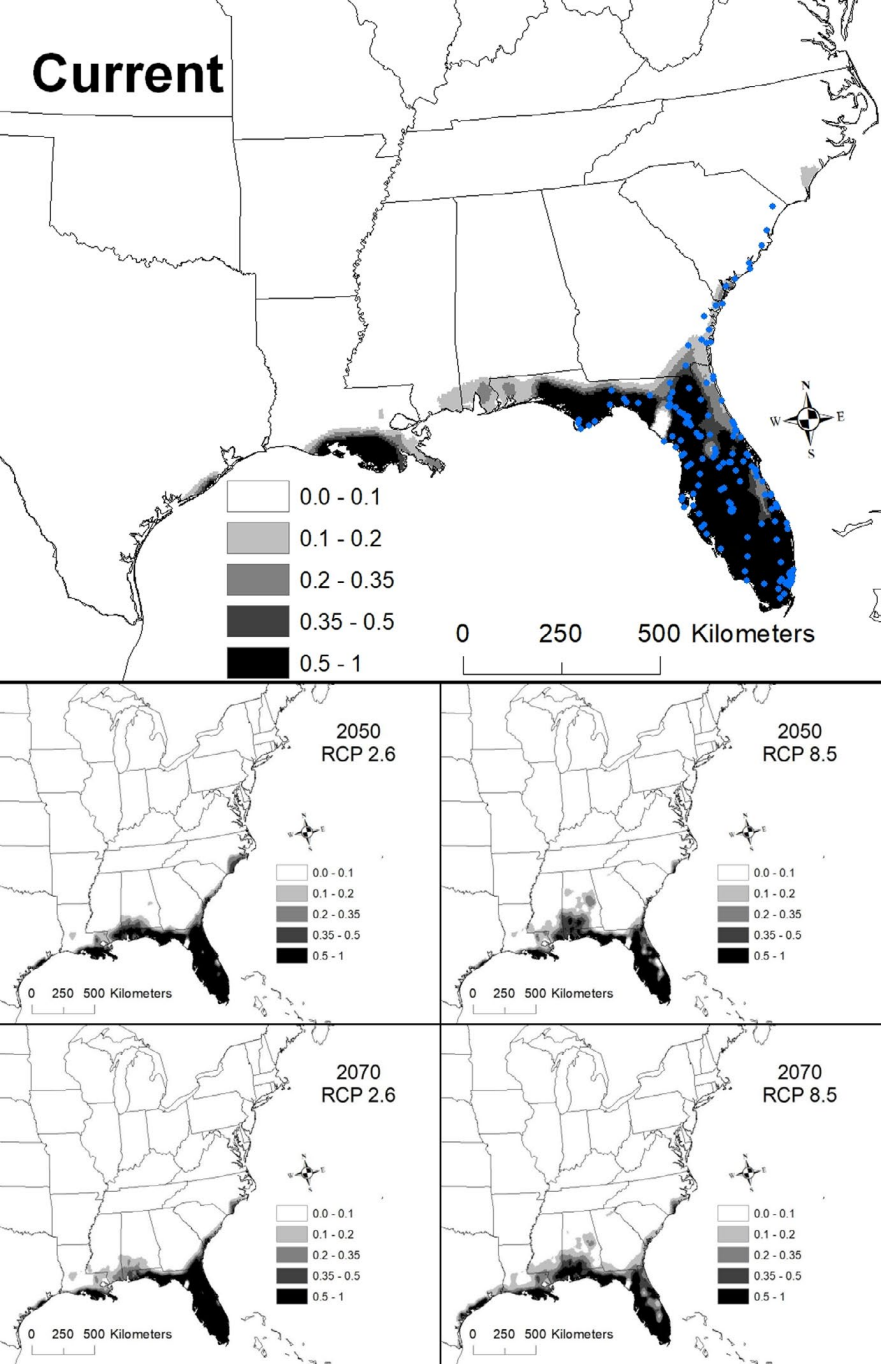
The modeled current and future distributions for *Sabal palmetto*. The legend shows the probability of occurrence, with the darkest shade representing >0.5 probability. Blue circles represent sites where *S. palmetto* (*n = *142) were located

The best models for *Sabal palmetto* included mean temperature of wettest quarter (BIO 8), mean temperature of coldest quarter (BIO 11), precipitation of wettest month (BIO 13), and precipitation of warmest quarter (BIO 18; Table 2). The AUC for this model was 0.987 ± 0.002. There was also some model support for precipitation of wettest quarter (BIO 16; Table 2). Areas that were predicted to have suitability >50% had a mean temperature of wettest quarter from 26.7 to 27.8°C, mean temperature of the coldest quarter >14.5°C, mean precipitation of the wettest month of 18.1–24.4 cm, and precipitation of warmest quarter >50.1 cm. Areas that are currently shown as >50% suitable were primarily in Florida, although a disjunct area of high suitability was also present in southern Louisiana (Figure 5).

**Figure 6:**
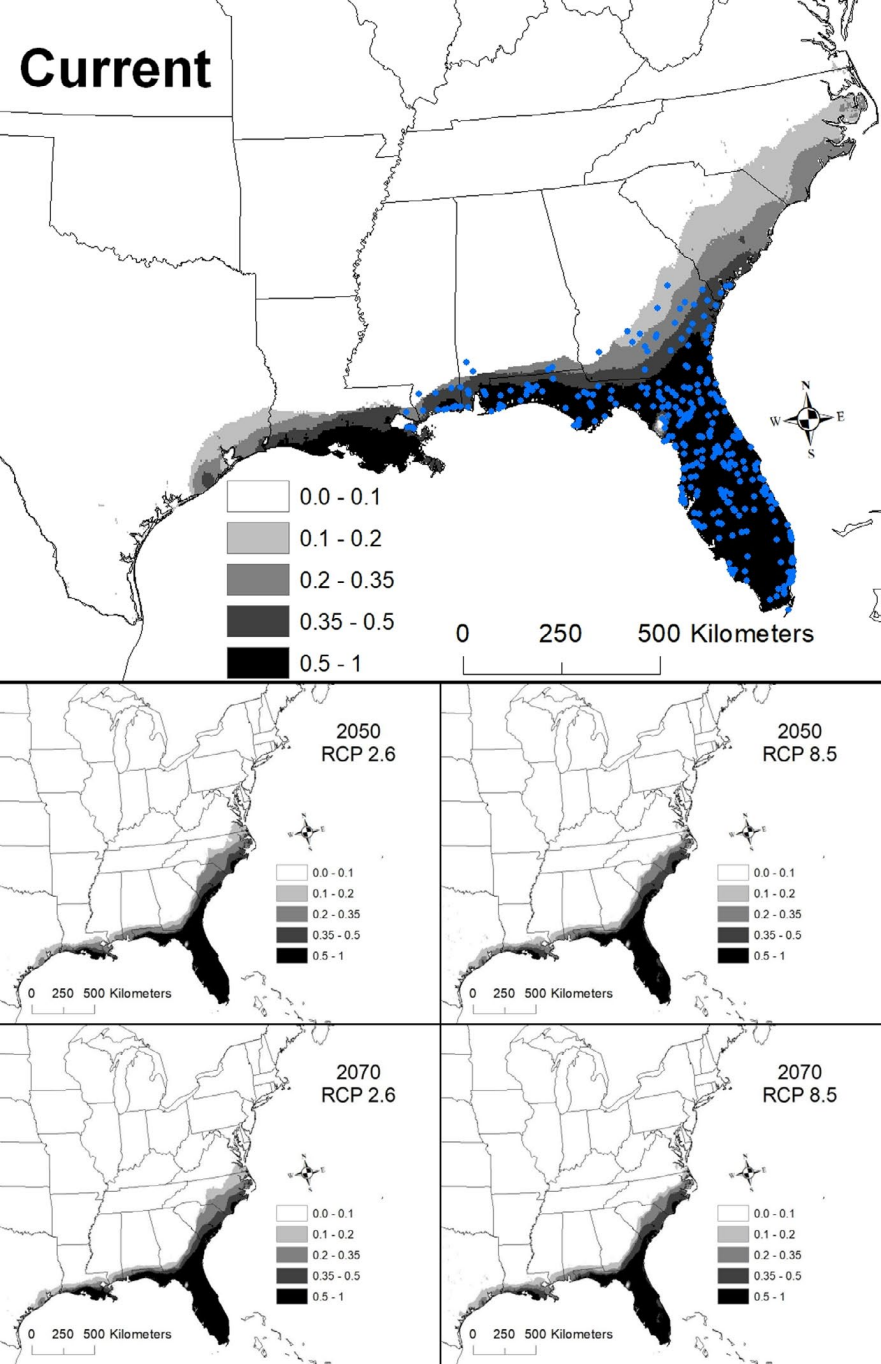
The modeled current and future distributions for *Serenoa repens*. The legend shows the probability of occurrence, with the darkest shade representing >0.5 probability. Blue circles represent sites where *S. palmetto* (*n = *298) were located

The best model for *Serenoa repens* included the variables minimum temperature of the coldest month (BIO 6), mean temperature of wettest quarter (BIO 8), precipitation of warmest quarter (BIO 18), and pH (Table 2). The AUC for this model was 0.977 ± 0.003. There was also some model support for mean temperature of coldest quarter (BIO 11) and precipitation of wettest quarter (BIO 16). Areas that were predicted to have suitability >50% had a minimum temperature of coldest month above 6.4°C, with a mean temperature of wettest quarter ranging from 26.5 to 27.8°C, precipitation of the warmest quarter above 46.7 cm, and in soils with a pH below 5.6. Areas that are currently shown as >50% suitability extended from southern Louisiana east to southern Georgia (Figure 6).

The median projected change in highly suitable conditions (i.e., those >50% suitability) for all five species by 2070 was −2% (range −99% to 30%), although there was considerable variation among species (Table 3). The area of highly suitable conditions for *Sabal minor* and *S. etonia* declined, while the area of highly suitable conditions for *Rhapidophyllum hystrix* and *Serenoa repens* remained largely unchanged, and the area for *Sabal palmetto* increased. However, the median amount of currently highly suitable conditions retained in future projections for these five species by 2070 was only 86% (range 1%–98%).

**Table 3:** The area forecast to have >50% probability of suitable conditions for each species under each climate change scenario

Species	Scenario	Area (km^2^)	% change in area	Area common to current (km^2^)	% current distribution retained
*Rhapidophyllum hystrix*	Current	280,324			
	2050 ‐ RCP 2.6	327,837	16.95	276,591	98.67
	2050 ‐ RCP 4.5	334,293	19.25	278,837	99.47
	2050 ‐ RCP 6.0	274,097	−2.22	261,485	93.28
	2050 ‐ RCP 8.5	271,674	−3.09	255,241	91.05
	2070 ‐ RCP 2.6	306,051	9.18	276,087	98.49
	2070 ‐ RCP 4.5	288,143	2.79	268,902	95.93
	2070 ‐ RCP 6.0	245,837	−12.30	234,973	83.82
	2070 ‐ RCP 8.5	274,749	−1.99	257,634	91.91
*Sabal etonia*	Current	86,194			
	2050 ‐ RCP 2.6	116,638	35.32	82,408	95.61
	2050 ‐ RCP 4.5	120,451	39.74	81,844	94.95
	2050 ‐ RCP 6.0	122,050	41.60	84,565	98.11
	2050 ‐ RCP 8.5	116,176	34.78	83,884	97.32
	2070 ‐ RCP 2.6	84,273	−2.23	78,618	91.21
	2070 ‐ RCP 4.5	44,591	−48.27	44,030	51.08
	2070 ‐ RCP 6.0	40,965	−52.47	39,841	46.22
	2070 ‐ RCP 8.5	15,109	−82.47	15,048	17.46
*Sabal minor*	Current	563,364			
	2050 ‐ RCP 2.6	154,368	−86.77	74,541	13.23
	2050 ‐ RCP 4.5	134,248	−88.88	62,647	11.12
	2050 ‐ RCP 6.0	113,234	−92.61	41,647	7.39
	2050 ‐ RCP 8.5	80,007	−97.42	14,554	2.58
	2070 ‐ RCP 2.6	171,217	−85.11	83,864	14.89
	2070 ‐ RCP 4.5	83,304	−97.13	16,153	2.87
	2070 ‐ RCP 6.0	82,808	−97.16	15,990	2.84
	2070 ‐ RCP 8.5	30,319	−98.82	6,660	1.18
*Sabal palmetto*	Current	134,250			
	2050 ‐ RCP 2.6	175,064	30.40	133,022	99.09
	2050 ‐ RCP 4.5	201,638	50.20	133,830	99.69
	2050 ‐ RCP 6.0	158,364	17.96	129,053	96.13
	2050 ‐ RCP 8.5	136,447	1.64	117,438	87.48
	2070 ‐ RCP 2.6	174,198	29.76	132,110	98.41
	2070 ‐ RCP 4.5	166,543	24.05	130,453	97.17
	2070 ‐ RCP 6.0	131,568	−2.00	115,610	86.12
	2070 ‐ RCP 8.5	157,948	17.65	114,006	84.92
*Serenoa repens*	Current	176,529			
	2050 ‐ RCP 2.6	177,411	0.50	150,975	85.52
	2050 ‐ RCP 4.5	199,447	12.98	162,287	91.93
	2050 ‐ RCP 6.0	172,470	−2.30	148,552	84.15
	2050 ‐ RCP 8.5	160,926	−8.84	139,878	79.24
	2070 ‐ RCP 2.6	194,420	10.14	165,200	93.58
	2070 ‐ RCP 4.5	211,350	19.73	162,146	91.85
	2070 ‐ RCP 6.0	185,738	5.22	150,682	85.36
	2070 ‐ RCP 8.5	181,107	2.59	145,926	82.66

The effect of climate change on suitable conditions for *Rhapidophyllum hystrix* varied depending on the scenario and year. For example, suitable conditions by 2050 expanded under scenario RCP 2.6, with highly suitable areas extending contiguously from southern Louisiana east to Florida and north to North Carolina (Figure 2). In contrast, a slight decline in suitability appears in Florida under the RCP 8.5 scenario by 2070 (Figure 2). A total of 280,324 km^2^ was identified as being currently highly suitable (i.e., modern‐day range with >50% chance of suitable environmental conditions). By 2050, the amount of highly suitable conditions ranged from 271,674 to 334,293 km^2^, of which 91%–99% was shared with the current model (Table 3). By 2070, the amount of highly suitable conditions declined to 245,837–306,051 km^2^, of which 84%–98% was shared with the current model (Table 3).

Under all scenarios, suitable conditions for *Sabal etonia* expanded to occupy most of the Florida peninsula by 2050 and then contracted by 2070 (Figure 3). A total of 86,194 km^2^ was identified as being currently highly suitable. By 2050, the amount of suitable areas increased substantially, ranging from 116,176 to 122,050 km^2^ of which 95%–98% was shared with the current model (Table 3). By 2070, the amount of highly suitable area declined to 15,109–84,272 km^2^, of which 17%–91% was shared with the current model (Table 2).

**Figure 7:**
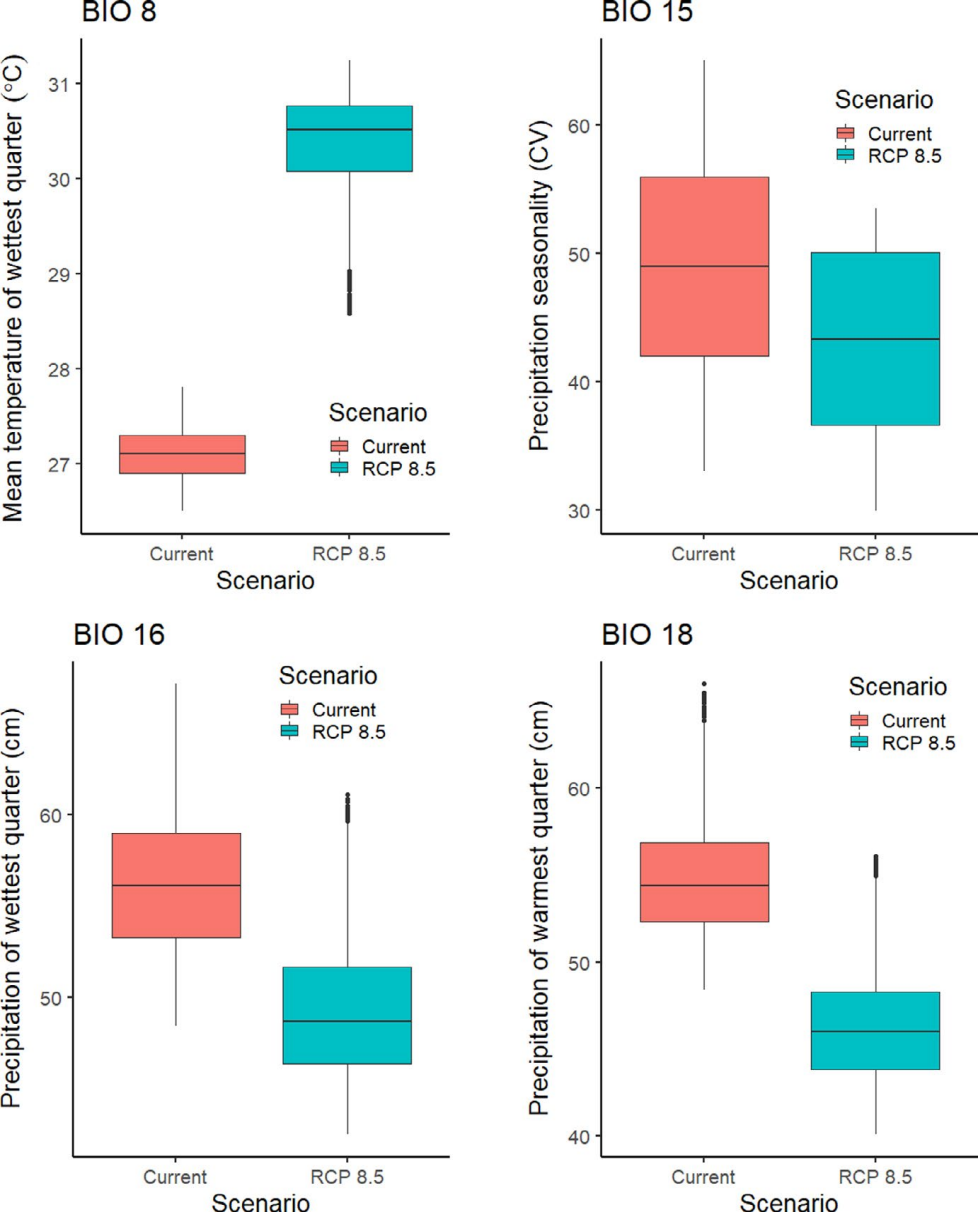
Boxplots of BIO 8 (mean temperature of wettest quarter), BIO 15 (coefficient of variation for precipitation seasonality), BIO 16 (precipitation of wettest quarter), and BIO 18 (precipitation of warmest quarter) for the area that currently has >0.5 probability of occurrence for *S. etonia,* showing the changes in bioclimatic variables in that area by 2070 under the RCP 8.5 scenario

The most severe decline in suitable conditions for *S. etonia* was under the 2070 RCP 8.5 scenario, which resulted in a reduction in suitable areas by 82%. This decline was associated with an increase in the mean temperature of the wettest quarter. The area currently identified as highly suitable for *Sabal etonia* has a median temperature of the wettest quarter of 27.1°C, but by 2070 under the RCP 8.5 scenario, this temperature had increased to 30.5°C (range 28.6–31.2°C; Figure 7). There was also a decline in projected precipitation of wettest quarter and precipitation of warmest quarter, although precipitation seasonality remained largely the same (Figure 7).

Under all scenarios, highly suitable conditions (i.e., >50%) for *S. minor* shifted toward the East Coast and the extent of highly suitable conditions declined (Figure 4). A total of 563,364 km^2^ was identified as being currently highly suitable. By 2050, the amount of suitable areas declined precipitously, ranging from 80,007 to 154,368 km^2^, and only 3%**–**13% of the highly suitable areas during 2050 was shared with the current model (Table 3). By 2070, the amount of highly suitable habitat declined further, ranging from 30,319 to 171,217 km^2^, of which only 1%–15% was shared with the current model (Table 3).

**Figure 8:**
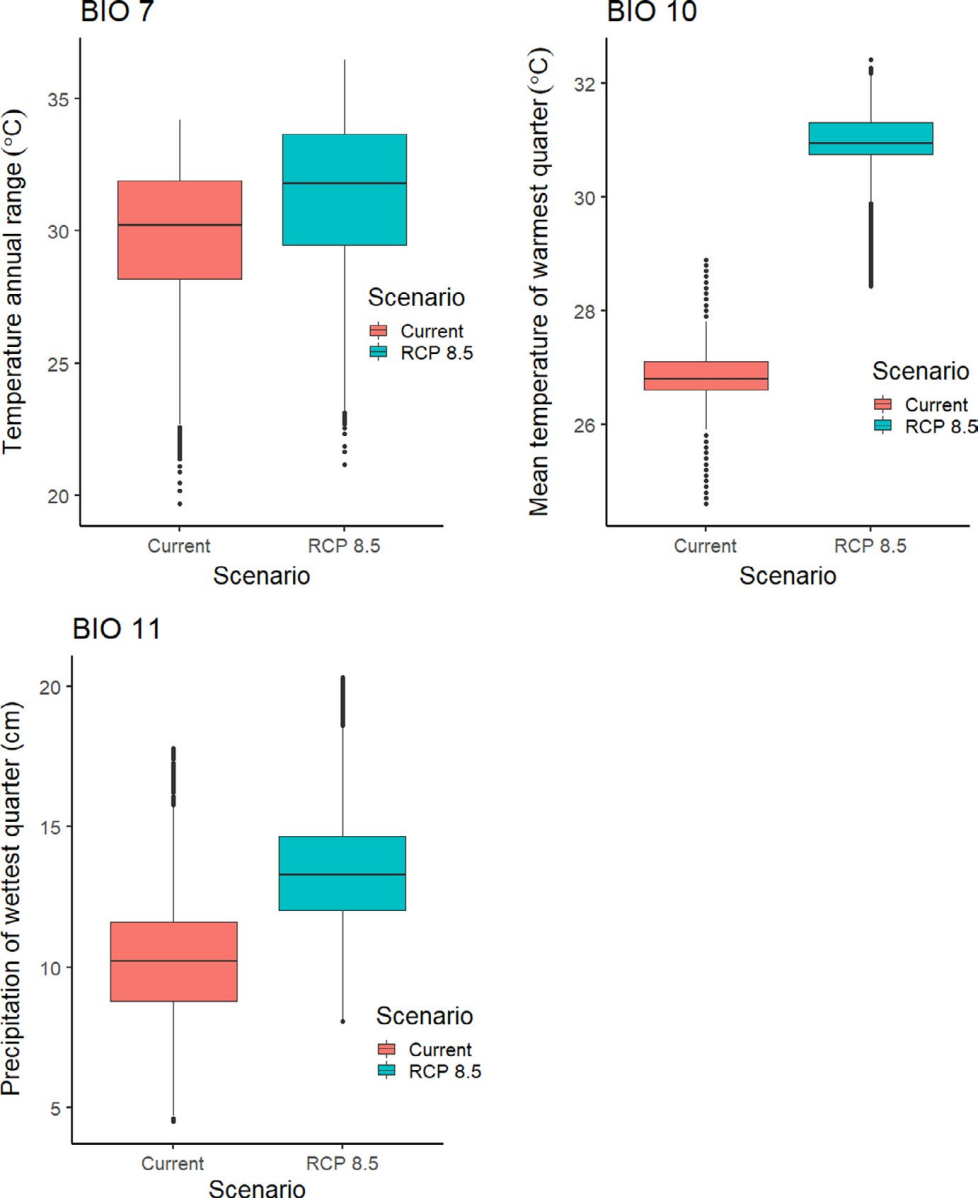
Boxplots of BIO 7 (temperature annual range), BIO 10 (mean temperature of warmest quarter), and BIO 11 (mean temperature of coldest quarter) for the area that currently has >0.5 probability of occurrence for *S. minor*, showing the changes in bioclimatic variables in that area by 2070 under the RCP 8.5 scenario

The most severe decline in extent for *S. minor* was under the 2070 RCP 8.5 scenario, which resulted in a decline of suitable areas by 99%. This decline was associated with an increase in the mean temperature of the warmest quarter. The area currently identified as highly suitable for *S. minor* has a median temperature of the warmest quarter of 26.8°C, but by 2070 under the RCP 8.5 scenario, this temperature had increased to 31.0°C (range 28.4–32.4°C; Figure 8).

Under nearly all scenarios, highly suitable habitat for *S. palmetto* expanded and shifted slightly northwest (Figure 5). A total of 134,250 km^2^ was identified as being currently highly suitable. Under all four of the 2050 scenarios and three of the four 2070 scenarios, the amount of highly suitable habitat increased, ranging from 136,447 to 201,638 km^2^, of which 85%–100% was shared with the current model (Table 3). However, under the 2070 RCP 6.0 scenario, the amount of highly suitable habitat decreased by 2% to 131,568 km^2^, of which 86% was shared with the current range (Table 3).

Under all scenarios, suitable conditions for *Serenoa repens* shifted slightly to the northeast and generally increased in extent (Figure 6). The change in distribution for *Serenoa repens* ranged from a decline of 9% to an increase of 20%, depending upon the scenario considered. A total of 176,529 km^2^ was identified as being currently highly suitable. By 2050, the area of suitable conditions contracted 2%–9% under the RCP 6.0 and RCP 8.5 scenarios, but increased by 1%–13% under the RCP 2.6 and RCP 4.5 scenarios (Table 3) By the 2070s, however, all scenarios resulted in an expansion of suitable conditions by 3%–20% (181,107–211,350 km^2^), of which 83%–94% was shared with the current range (Table 3).

**Figure 9:**
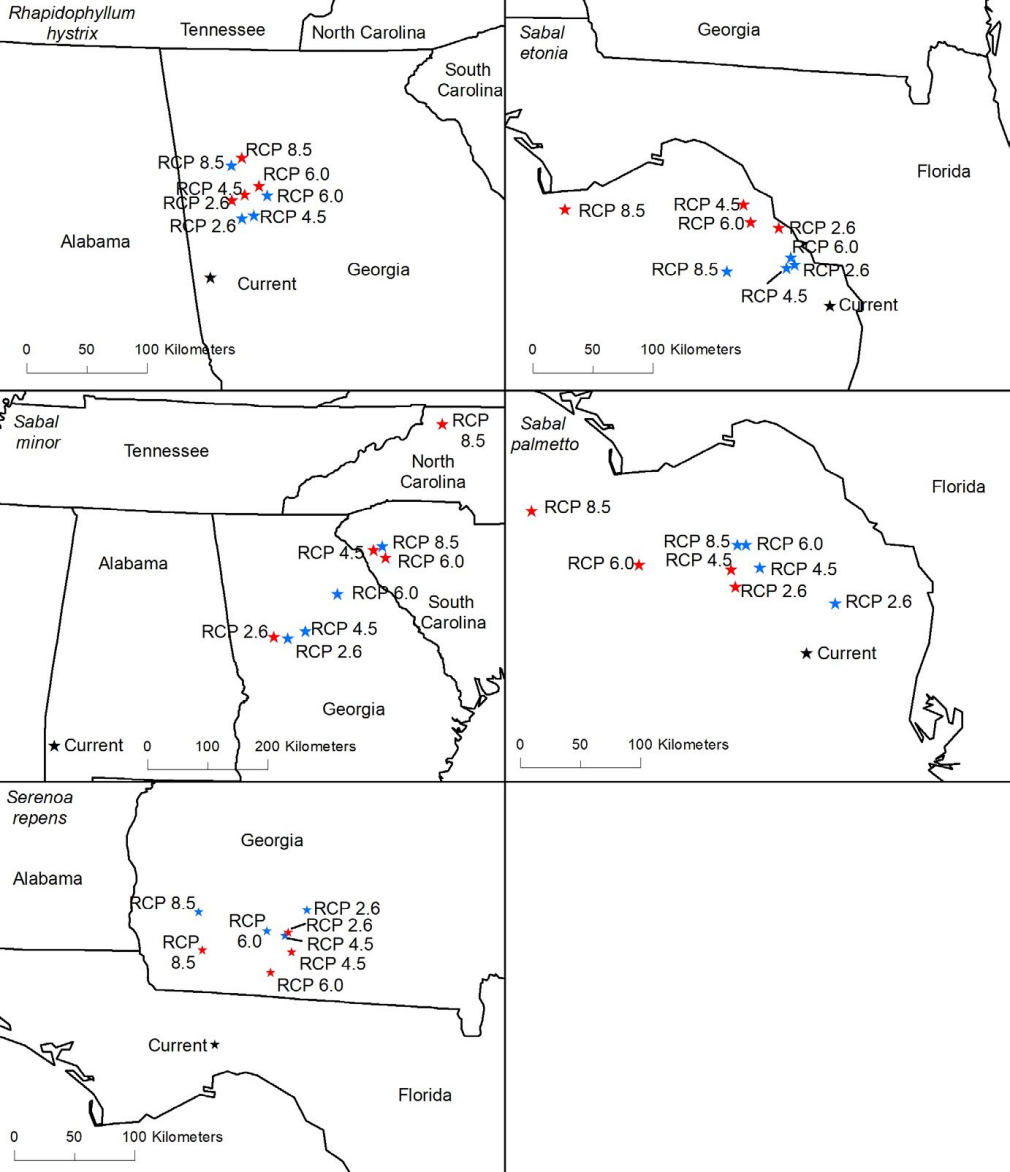
The centroids are the geometric center of the range of each species under each scenario. A black star represents the current centroid, while blue stars show projected centroids by 2050 and red stars show projected centroids by 2070. Due to the concave distribution of *Sabal etonia* and *S. palmetto*, centroids for these species are present in the Gulf of Mexico, where neither are expected to occur

Centroids shifted generally northward for each of the five species (Figure 9), at a median rate of 23.5 km/decade. However, the response rate varied substantially among species. Under all scenarios, the rate of change for *Rhapidophyllum hystrix* was 11–24 km/decade (Table 4). Centroids for *Serenoa repens* and *S. palmetto* shifted at a moderate rate of 13–34 km and 12–43 km/decade, respectively. However, centroids for *S. minor* shifted much faster (68–160 km/decade) than other species. The rapid shift to the northeast for *S. minor* centroids should be interpreted cautiously, however, because a pronounced range contraction is forecast resulting in greater weights being assigned to locations currently beyond the natural range of this species.

**Table 4:** The distance from the centroid for each scenario to the current centroid as well as the rate per decade

Species	Scenario	Distance (km) and direction to current	Rate per decade (km/decade)
*Rhapidophyllum hystrix*	2050 ‐ RCP 2.6	56 (NNE)	14
	2050 ‐ RCP 4.5	63 (NE)	21
	2050 ‐ RCP 6.0	83 (NE)	16
	2050 ‐ RCP 8.5	95 (NNE)	24
	2070 ‐ RCP 2.6	67 (NNE)	11
	2070 ‐ RCP 4.5	75 (NNE)	13
	2070 ‐ RCP 6.0	87 (NNE)	15
	2070 ‐ RCP 8.5	103 (NE)	17
*Sabal etonia*	2050 ‐ RCP 2.6	45 (NW)	11
	2050 ‐ RCP 4.5	48 (NW)	12
	2050 ‐ RCP 6.0	52 (NW)	13
	2050 ‐ RCP 8.5	90 (WNW)	23
	2070 ‐ RCP 2.6	78 (NW)	20
	2070 ‐ RCP 4.5	111 (NW)	28
	2070 ‐ RCP 6.0	96 (NW)	24
	2070 ‐ RCP 8.5	234 (WNW)	59
*S. minor*	2050 ‐ RCP 2.6	429 (NE)	107
	2050 ‐ RCP 4.5	461 (NE)	115
	2050 ‐ RCP 6.0	536 (NE)	134
	2050 ‐ RCP 8.5	641 (NE)	160
	2070 ‐ RCP 2.6	409 (NE)	68
	2070 ‐ RCP 4.5	625 (NE)	104
	2070 ‐ RCP 6.0	636 (NE)	106
	2070 ‐ RCP 8.5	840 (NE)	140
*S. palmetto*	2050 ‐ RCP 2.6	48 (NNE)	12
	2050 ‐ RCP 4.5	82 (NW)	21
	2050 ‐ RCP 6.0	104 (NW)	26
	2050 ‐ RCP 8.5	107 (NW)	27
	2070 ‐ RCP 2.6	81 (NW)	14
	2070 ‐ RCP 4.5	94 (NW)	16
	2070 ‐ RCP 6.0	158 (WNW)	26
	2070 ‐ RCP 8.5	257 (WNW)	43
*Serenoa repens*	2050 ‐ RCP 2.6	136 (NE)	34
	2050 ‐ RCP 4.5	108 (NE)	27
	2050 ‐ RCP 6.0	104 (NNE)	26
	2050 ‐ RCP 8.5	111 (NNW)	28
	2070 ‐ RCP 2.6	112 (NE)	19
	2070 ‐ RCP 4.5	100 (NE)	17
	2070 ‐ RCP 6.0	76 (NE)	13
	2070 ‐ RCP 8.5	79 (NNW)	13

## 4 DISCUSSION

The modeled current ranges for the five palm species closely mirrored published range maps (Henderson et al., 1995; Zona, 2000), although we projected potentially suitable habitat outside of the current natural range for some species. For example, models of *Rhapidophyllum hystrix* and *S. palmetto* predicted suitable habitat in southern Louisiana, where neither species is native. MaxEnt is effective at identifying the potential distribution of plants in new environments (e.g., Wilson et al., 2009) and so it is unsurprising that potentially suitable habitat was identified beyond the established range. In this case, southern Mississippi and Alabama exhibit colder winter minima than southern Louisiana or Florida (USDA, 2012), which may have acted as a barrier to western dispersal. Accordingly, it is unsurprising that the native range of *Rhapidophyllum hystrix* and *S. palmetto* does not extend to southern Louisiana, although *S. palmetto* and *Rhapidophyllum hystrix* are widely planted in this area (pers. obs.). Additionally, *S. palmetto* may have been more widespread during the Pleistocene, as there are substantial numbers of a hybrid between *S. minor* and *S. palmetto* (*Sabal x brazoriensis* D.H. Goldman, L. Lockett, & R.W. Read, nothosp. nov.) in Brazoria County, Texas, which is located approximately 1,000 km west of the nearest native *Sabal palmetto* population (Goldman, Klooster, & Griffith, 2011).

Palms are commonly used as indicators for megathermal climates (e.g., Pross et al., 2012; Reichgelt, West, & Greenwood, 2018) and therefore should be especially responsive to climate change. Our models suggest that highly suitable habitat for *S. etonia* and *S. minor* will decline substantially in extent during the 21st century while the amount of highly suitable habitat for *Rhapidophyllum hystrix* will stay largely constant. Highly suitable habitat is projected to slightly increase for *Serenoa repens* and substantially increase for *S. palmetto*. These results broadly mirror the results published on other taxa in the southeastern United States, which show some species increasing in extent while other species decline under anthropogenic climate change (e.g., Butler et al., 2016; McKenney, Pedlar, Lawrence, Campbell, & Hutchinson, 2007; Osland, Enwright, Day, & Doyle, 2013).

Despite the potential for the range of some palm species to increase in extent, these five species may be unable to enlarge their ranges as rapidly as the habitat becomes potentially suitable. For example, by 2050, the extent of highly suitable habitat for *S. palmetto* is projected to increase by 18%–50%. However, the most frequent dispersal method of *S. palmetto* seeds is by raccoon (*Procyon lotor*), gopher tortoise (*Gopherus polyphemus*), white‐tailed deer (*Odocoileus virginianus*), and feral hog (*Sus scrofa*; Abrahamson & Abrahamson, 1989), none of which typically disperse very far (Gehrt & Fritzell, 1998; Kilgo, Labisky, & Fritzen, 1996; McRae, Landers, & Garner, 1981; Truvé & Lemel, 2003). Although birds may also occasionally feed on *S. palmetto* seeds, fruit set is during October when many bird species are migrating south (Stiles, 1980), which makes it unlikely that avian frugivory will facilitate northward dispersal. Additionally, in northern Florida, it takes a minimum of 14 years for wild *Sabal palmetto* to begin growing a trunk and 59 years for half of all individuals to develop a trunk (McPherson & Williams, 1996). Since *S. palmetto* will not fruit until it has developed a trunk (Fox & Andreu, 2019), expansion of *S. palmetto* by animal dispersal outside of its current range is likely to be slow.

Likewise, the potential for *Serenoa repens* to rapidly colonize new suitable habitat appears to be limited. While *Serenoa repens* produces viable seeds and seedlings, in Florida it appears to spread primarily by vegetative sprouts, with some genets speculated to have been present for millennia (Takahashi, Horner, Kubota, Keller, & Abrahamson, 2011). Although seedlings exhibit relatively high survivorship, with 35%–57% surviving over a 19‐year study, average growth rate is very slow and was generally <0.5 cm per year (Abrahamson & Abrahamson, 2009), although the growth rate of some individuals may be higher in the absence of exotic grasses (Foster & Schmalzer, 2012). The combination of primarily vegetative spread and a very slow growth rate suggests that the ability of this species to expand its range in concordance with the changing climate is probably extraordinarily low.

However, the models for the projected distributions of *Serenoa repens* should be interpreted cautiously. Although *Serenoa repens* is endemic to the United States, the native range extends to the southern tip of Florida (Henderson et al., 1995). Consequently, it is possible that the environmental variables considered in this study do not represent the full range of environmental conditions it could tolerate and reproduce in. For example, *Serenoa repens* is present on Key West National Wildlife Refuge (Florida), and it is plausible that *Serenoa repens* could successfully grow in Cuba, as the distance from Havana (Cuba) to Key West, Florida (USA), is only approximately 170 km. Consequently, it is conceivable that *Serenoa repens* could potentially tolerate more tropical conditions than this study considered.

We projected that the extent of highly suitable habitat for *S. minor* will exhibit a dramatic decline during the 21st century. The current distribution of the dwarf palmetto, *S. minor*, extends from Oklahoma to Texas and east to North Carolina and Florida (Butler & Tran, 2017). Globally, it is listed as a secure species, and at the state level, it is not considered to be a species of special concern across most of its range, with the exception of North Carolina where it is listed as S3 (Vulnerable) species and Oklahoma where it is listed as a S2 (Imperiled) (NatureServe, 2019; ONHI, 2017). However, we project that the amount of highly suitable habitat for *S. minor* will decline by 87%–93% by 2050, driven primarily by an increase in the mean temperature of the warmest quarter across its current range. It is conceivable that *S. minor* may be able to withstand warmer temperatures than current conditions. For example, Goldman (1999) documented an isolated population of *S. minor* south of the main range in Nuevo León, Mexico (Goldman, 1999). However, this population shows introgression with *S. mexicana* (Goldman et al., 2011), a species that is widespread in Central America, and it is possible that the tolerance of *S. minor* for the climate in this location could be partly genetic.

The median projected centroid shift for each species was 23.5 km/decade and ranged from 11 to 160 km/decade. However, palms typically exhibit low dispersal ability (Bacon et al., 2013), and it may not be possible for these species to expand their range at this rate. Animal seed dispersers are an important component of palm reproduction (Zona & Henderson, 1989), and seed dispersal for the five study species is primarily by animals (Zona, 2006) although information on seed dispersal in some of the five palm species is very limited. For example, the only documented animal dispersing seeds of *Rhapidophyllum hystrix* are the black bear (*Ursus americanus*; Maehr, 1984). Additionally, anthropogenic climate change may affect plant recruitment and could potentially enhance, delay or even preclude seed regeneration (Walck, Hidayati, Dixon, Thompson, & Poschlod, 2011). Furthermore, we did not incorporate the sea level rise of 0.5–1.4 m above 1990 levels, projected to occur by the end of the 21st century (Rahmstorf, 2007), which may potentially reduce the extent of suitable habitat for all five palms. Finally, we did not attempt to incorporate changes in land use in our models, which may affect the prevalence of palms in the future. For example, both *S. etonia* and *Serenoa repens* exhibit strong flowering responses after episodic fires (Abrahamson, 1999; Carrington & Mullahey, 2013). Efforts to suppress fires, therefore, could potentially restrict the persistence of these species on the landscape during the coming decades.

However, some species may disperse in a fashion that leads to isolated founder plants that can establish new populations, if local environmental conditions are suitable (Shapcock et al., 2020). Given that four of the five species considered here are common in the nursery trade, it is possible that individuals planted in gardens outside of the native range may act facilitate naturalization for future generations, similar to the pattern observed for *Trachycarpus fortunei* in Switzerland (Fehr & Burga, 2016) and eight invasive palm species in Panama (Svenning, 2002).

In addition to changes in temperature, precipitation, and seasonality, ongoing increases in atmospheric greenhouse gases are affecting growth and physiology in plants (Thompson, Gamage, Hirotsu, Martin, & Seneweera, 2017). Increasing levels of CO_2_ have increased growing season leaf area, particularly in the tropics (Zhu et al., 2016). In addition, elevated CO_2_ levels have been linked to increased water use efficiency (Keenan et al., 2013), photosynthesis (Lee, Barrott, & Reich, 2011), root growth (Wang et al., 2009), and stem growth (Burgess & Huang, 2014). It seems likely that elevated CO_2_ will likewise be beneficial to palm growth, development, and physiology (Henson & Harun, 2005; Ibraham & Jaafar, 2012).

Overall, however, the ability of these five palm species to take advantage of suitable conditions outside of their native range appears to be limited. Additionally, Lavergne, Mouquet, Thuiller, and Ronce (2010) suggested the long‐lived species with low rates of reproduction and dispersal may not be able to keep pace with environmental changes wrought by anthropogenic climate change. Native palm species in the southeastern United States appear to fit this mold, as they exhibit high adult survivorship coupled with a low dispersal ability. *Sabal minor*, for example, may reach up 400 years of age (Ramp, 1989) and individual stems of *Serenoa repens* may live to 700 years with near‐zero annual mortality (Abrahamson, 1995). However, palms can also exhibit physiological methods for dealing with unfavorable conditions, including heat and drought (Abrahamson & Abrahamson, 2002; Arab et al., 2016; Renninger & Phillips, 2016). Consequently, while conditions in current native range may become increasingly unsuitable for some species, these palms may temporarily avoid local extinction, particularly if they are able to take advantage of refugia (Ashcroft, Chisholm, & French, 2009; McLaughlin et al., 2017). Nonetheless, these responses will likely be insufficient to prevent local extinction over the long term.

## CONFLICT OF INTEREST

There are no competing interests.

## AUTHOR CONTRIBUTION

**Christopher Butler:** Conceptualization (lead); Data curation (supporting); Formal analysis (lead); Funding acquisition (lead); Investigation (equal); Methodology (equal); Project administration (lead); Resources (equal); Validation (lead); Visualization (lead); Writing‐original draft (lead); Writing‐review & editing (equal). **Matt Larson:** Data curation (lead); Formal analysis (supporting); Investigation (equal); Methodology (equal); Visualization (supporting); Writing‐original draft (supporting); Writing‐review & editing (equal).

## Data Availability

Locality data are archived and openly available at the Harvard Dataverse system (http://dataverse.harvard.edu/) at https://doi.org/10.7910/DVN/RXML2U
